# FL% is associated with the severity of acute DeBakey type I aortic dissection in patients undergoing frozen elephant trunk and total arch replacement

**DOI:** 10.3389/fsurg.2024.1329771

**Published:** 2024-04-09

**Authors:** Long-Fei Wang, Yu Li, Mu Jin, Hai-Bin Li, Nan Zhang, Ming Gong, Hong-Jia Zhang, Yu-Yong Liu, Yong-Qiang Lai

**Affiliations:** ^1^ Department of Cardiovascular Surgery, Beijing Anzhen Hospital, Capital Medical University, Beijing, China; ^2^ Department of Radiology, The Seventh Affiliated Hospital, Sun Yat-sen University, Shenzhen, China; ^3^ Department of Anesthesiology, Beijing Friendship Hospital, Capital Medical University, Beijing, China; ^4^ Department of Cardiac Surgery, Beijing Chaoyang Hospital, Capital Medical University, Beijing, China; ^5^ Department of Radiology, Beijing Anzhen Hospital, Capital Medical University, Beijing, China

**Keywords:** computed tomography angiography (CTA), risk factors, severity (S), acute, DeBakey type I aortic dissection

## Abstract

**Objectives:**

The diameter, area, and volume of the true lumen and false lumen (FL) have been measured in previous studies to evaluate the extent of DeBakey type I aortic dissection. However, these indicators have limitations because of the irregular shapes of the true and false lumens and the constant oscillation of intimal flap during systole and diastole. The ratio of arch lengths seems to be a more reliable indicator. FL% was defined as the ratio of the arch length of FL to the circumference of the aorta at the different levels of the aorta. The purpose of this article was to investigate whether FL% is a predictor of the severity of acute DeBakey type I aortic dissection in patients undergoing frozen elephant trunk (FET) and total arch replacement.

**Methods:**

In this retrospective observational study, we analyzed a total of 344 patients with acute DeBakey type I aortic dissection that underwent FET and total arch replacement at our center from October 2015 to October 2019. The patients were divided into two groups by cluster analysis according to the perioperative course. Binary logistic regression analyses were performed to determine whether FL% could predict the severity of acute DeBakey type I aortic dissection. The area under the receiver operating characteristic curve (AUROC) was used to assess the power of the multivariate logistic regression model for the severity of acute DeBakey type I aortic dissection.

**Results:**

The patients in the ultra-high-risk group (109 patients) had significantly more severe clinical comorbidities and complications than the patients in the high-risk group (235 patients). The ascending aortic FL% [odds ratio (OR), 11.929 (95% CI: 1.421–100.11); *P* = 0.022], location of initial tear [OR, 0.68 (95% CI: 0.47–0.98); *P* = 0.041], the degree of left iliac artery involvement [OR, 1.95 (95% CI: 1.15–3.30); *P* = 0.013], and the degree of right coronary artery involvement [OR, 1.46 (95% CI: 1.01–2.12); *P* = 0.045] on preoperative computed tomography angiography were associated with the severity of acute DeBakey type I aortic dissection. The AUROC value of this multivariate logistic regression analysis was 0.940 (95% CI: 0.914–0.967; *P* < 0.001). The AUROC value of ascending aortic FL% was 0.841 (95% CI: 0.798–0.884; *P* < 0.001) for the severity of acute DeBakey type I aortic dissection in patients undergoing FET and total arch replacement.

**Conclusions:**

Ascending aortic FL% was validated as an essential radiologic index for assessing the severity of acute DeBakey type I aortic dissection in patients undergoing FET and total arch replacement. Higher values of ascending aortic FL% were more severe.

## Introduction

Acute (≤14 days from symptom onset to surgery) DeBakey type I aortic dissection (AD) remains one of the most lethal cardiovascular diseases (1–4). Aortic computed tomography angiography (CTA) is the first choice and a reliable examination method to diagnose DeBakey type I aortic dissection (5, 6). AD is characterized by an entry tear in the aortic intima. The true and false lumens (FL) expand with intimal flap (7). The diameter, area, and volume of the true lumen and FL have been measured in previous studies to evaluate the extent of DeBakey type I aortic dissection (8–10). However, these indicators have limitations due to the irregular shapes of the true and false lumens and the constantly oscillating intimal flap.

**Figure 1:**
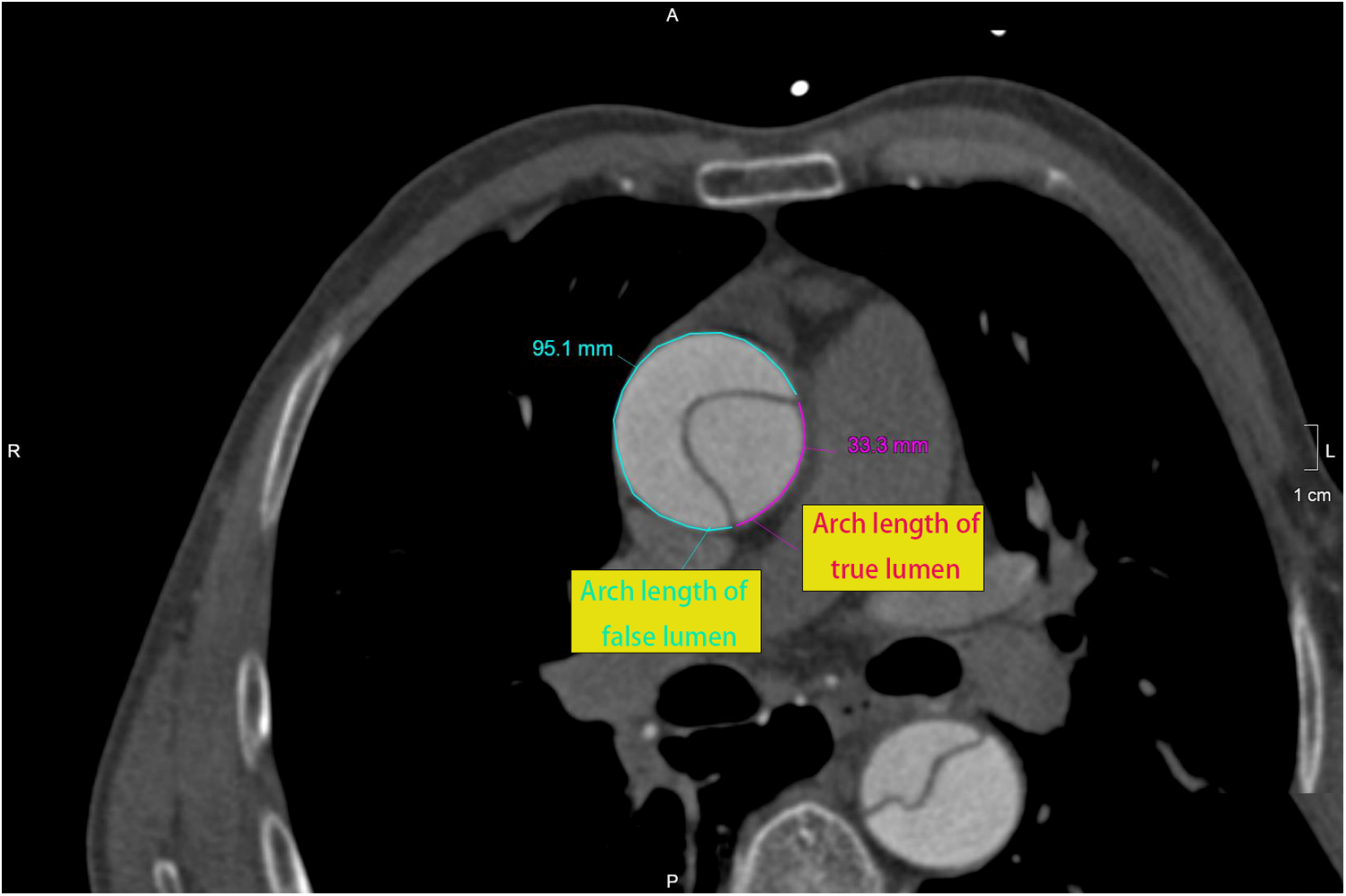
At different levels of the aorta, the arch length of false lumen (95.1 mm) and true lumen (33.3 mm) were measured at the ascending aorta (2 cm above the sino-tubular junction). FL% was 74.1% [95.1/(95.1 + 33.3)].

The ratio of arch lengths in DeBakey type I aortic dissection seems to be a more reliable indicator. FL% was defined as the ratio of FL arch length to aortic circumference at different aortic levels (Figure 1). The purpose of this article was to investigate whether FL% is a predictor of the severity of acute DeBakey type I aortic dissection in patients undergoing frozen elephant trunk (FET) and total arch replacement (11).

## Patients and methods

### Ethical statement

This study was approved by the Ethics Committee of the Beijing Anzhen Hospital (Institutional Review Board File 2023125X). Informed consent was waived from each patient due to the retrospective nature of this research and the anonymous storage of data. All patient information was kept confidential.

### Patients

From October 2015 to October 2019, 344 patients were enrolled in this study and retrospectively reviewed. Inclusion criteria included the following: (1) confirmation with DeBakey type I aortic dissection; (2) time from symptom onset to surgery ≤14 days; (3) age >18 years; (4) patients undergoing FET and total arch replacement techniques. The exclusion criteria were as follows: (1) no available preoperative aortic CTA data; (2) history of organ failure, coronary artery disease, or cerebral infarction; (3) pregnant women. Preoperative status, imaging data, and intraoperative and postoperative data related to the severity of acute DeBakey type I aortic dissection were reviewed and recorded. The mean age was 47.7 ± 11.0 years (range, 14–80), and 274 (79.7%) were male.

### Imaging

A second-generation dual-source CT scanner (Somatom Definition Flash; Siemens Healthineers, Germany) with a high-pitch spiral scan mode was used. CTA scanning was performed using the electrocardiogram-triggered flash protocol. All patients were placed in the supine position. Both arms were elevated. The anatomical range of CTA imaging was from above the aortic arch to the pubic bone in the cranio-caudal direction. CTA scan parameters were set as follows: tube voltage and current, 100 kV and 320 mAs, respectively; detector collimation, 2 mm × 64 mm × 0.6 mm; pitch, 3.4; and gantry rotation time, 0.28 s. A dose of 1 ml/kg contrast was injected at a rate of 4–5 ml/s, followed by 30 ml of saline. Image data were transferred to the workstation (Vital Images, Minnetonka, MN, USA) for postprocessing.

This analysis process of aortic CTA images for each patient was carried out by two trained radiological observers, using a double-blind method. The measurements of FL% were performed at five levels, including ascending aorta (2 cm above the sino-tubular junction), thoracic-descending aorta (the level of main pulmonary artery bifurcation), descending aorta (the level of diaphragm), and two levels of abdominal aorta (levels of celiac trunk and right renal artery). We defined AD% as the percentage of the length of the dissection to the whole length of the aorta. The range of the whole length of the aorta was from the aortic root to the bifurcation of the left and right common iliac arteries.

We defined the perfusion pattern for each branch as 1 (perfusion supplied by true lumen), 2 (perfusion supplied by true and false lumen), and 3 (perfusion supplied by false lumen), with reference to the branch perfusion classification proposed by Sukgu and the Penn classification (12, 13). The right coronary artery, left coronary artery, innominate artery, left carotid artery, left subclavian artery, celiac trunk, superior mesenteric artery, right renal artery, left renal artery, right common iliac artery, and left common iliac artery were analyzed.

### Data analysis and statistical methods

Statistical analysis was performed with the Statistical Package for the Social Sciences (SPSS) Statistics (version 26.0.0 for Mac OS; IBM). Quantitative variables were expressed as mean ± standard deviation or median and interquartile range for non-normal distribution. Categorical variables were expressed as frequencies and percentages. According to preoperative, intraoperative, and postoperative indicators, the participants were divided into two severity groups by two-step cluster analysis. The two groups were defined as ultra-high-risk group, with more severe clinical manifestations, and high-risk group by univariate analysis. The univariate analysis was performed using Student’s t test, Pearson chi-squared test, or Fisher exact test, as appropriate. Variables with *P* < 0.05 on the univariate analysis were evaluated by multivariate analysis using a forward stepwise binary logistic regression model. The area under the receiver operating characteristic curve (AUROC) was used to assess the power of the multivariate logistic regression model for the severity of acute DeBakey type I aortic dissection.

## Results

### Two severity grade groups by cluster analysis

The main variables that could reflect the severity of the DeBakey type I aortic dissection were recorded, including preoperative (Table 1), intraoperative (Table 2), and postoperative (Table 3) variables. The aforementioned variables were used for cluster analysis. The two-step cluster analysis showed that there were two clusters (cluster 1 with 109 patients and cluster 2 with 235 patients) in this cohort.

**Table 1:** Preoperative clinical profiles.

Variable	Cluster 1 (*n* = 109)	Cluster 2 (*n* = 235)	*P*-value
Age (years)	50.78 ± 10.12	45.99 ± 10.04	<0.001
Gender (male)	82 (75.2%)	192 (81.7%)	0.165
Stroke	6 (5.5%)	16 (6.8%)	0.646
AKI	0	0	—
Limb ischemia	16 (14.7%)	28 (11.9%)	0.341
Gastrointestinal bleeding	0	1 (0.4%)	>0.999
Spinal cord injury	6 (5.5%)	6 (2.6%)	0.284
Acute myocardial infarction	0	1 (0.4%)	>0.999
Aortic regurgitation	93 (85.3%)	169 (71.9%)	<0.001
None	16 (14.7%)	66 (28.1%)	0.007
Mild	39 (35.8%)	92 (39.1%)	0.549
Moderate	27 (24.8%)	50 (21.3%)	0.469
Serious	27 (24.8%)	27 (11.5%)	0.002
Pericardial effusion	26 (23.9%)	34 (14.5%)	0.026
None	83 (76.1%)	201 (85.5%)	0.033
Mild	18 (16.5%)	27 (11.5%)	0.238
Moderate	4 (3.7%)	7 (3.0%)	0.992
Serious	4 (3.7%)	0	0.016
Pleural effusion	29 (26.6%)	51 (21.7%)	0.309
None	80 (73.4%)	184 (78.3%)	0.317
Mild	28 (25.7%)	50 (21.3%)	0.363
Moderate	0	1 (0.4%)	>0.999
Serious	1 (0.9%)	0	0.317
LVEF	62.00 (58.00–66.00)	62.09 (60.00–66.00)	>0.999
D-dimer (ng/ml)	2,915.00 (1,717.50–10,836.50)	1,987.00 (918.00–2,875.00)	0.041
Myoglobin (ng/ml)	75.30 (31.00–208.60)	33.30 (20.00–66.40)	>0.999
Trop-I (ng/ml)	0.03 (0.01–0.21)	0.01 (0.00–0.04)	0.001
ALT (U/L)	24.00 (18.00–43.00)	22.00 (16.00–33.00)	0.031
AST (U/L)	31.00 (20.00–48.50)	22.00 (18.00–27.00)	<0.001
Creatinine (μmol/L)	86.90 (74.85–122.35)	79.20 (67.30–95.80)	<0.001

**Table 2:** Intraoperative data.

Variable	Cluster 1 (*n* = 109)	Cluster 2 (*n* = 235)	*P*-value
Operative time (h)	8.10 (7.30–9.20)	7.20 (6.50–8.00)	<0.001
CPB time (min)	240.48 ± 57.98	201.00 (180.00–220.00)	<0.001
Crossclamp time (min)	134.73 ± 36.75	109.00 (95.00–128.00)	<0.001
Selective cerebral perfusion time (min)	38.02 ± 12.23	35.00 (29.00–41.00)	0.036
Concomitant procedures			
Ascending aortic replacement	109 (100%)	235 (100%)	>0.999
Aortic root procedures			
Repair of sinus of Valsalva	15 (13.8%)	5 (2.1%)	<0.001
David procedure	6 (5.5%)	2 (0.9%)	0.023
Wheat procedure	6 (5.5%)	4 (1.7%)	0.108
Cabrol procedure	3 (2.7%)	2 (0.9%)	0.381
Bentall procedure	73 (67%)	30 (12.8%)	<0.001
Other concomitant procedures			
Coronary artery bypass grafting	24 (22%)	10 (4.3%)	<0.001
Mitral valve operation	1 (0.9%)	3 (1.3%)	>0.999
Extra-anatomic bypass	12 (11%)	5 (2.1%)	<0.001

**Table 3:** Postoperative data.

Variable	Cluster 1 (*n* = 109)	Cluster 2 (*n* = 235)	*P*-value
Duration of intubation (h)	64.00 (29.50–133.50)	16.00 (12.00–34.00)	<0.001
ICU length of stay (h)	134.00 (44.50–238.00)	39.00 (23.00–66.00)	<0.001
Hospitalization cost (￥)	245,617.70 (201,982.66–300,891.46)	172,906.93 ± 32,437.72	<0.001
Spinal cord injury	9 (8.3%)	1 (0.4%)	<0.001
Low cardiac output syndrome	17 (15.6%)	0	<0.001
Stroke	18 (16.5%)	3 (1.3%)	<0.001
Pulmonary complications	34 (31.2%)	0	<0.001
Acute renal failure	23 (21.1%)	0	<0.001
Gastrointestinal complications	14 (12.8%)	3 (1.3%)	<0.001
Death	24 (22.0%)	2 (0.9%)	<0.001

The preoperative indicators included stroke, acute kidney injury (AKI), limb ischemia, gastrointestinal bleeding, spinal cord injury, acute myocardial infarction, aortic regurgitation, pericardial effusion, left ventricular ejection fraction (LVEF), pleural effusion, plasma levels of D-dimer, myoglobin, troponin I (Trop-I), alanine aminotransferase (ALT), aspartate aminotransferase (AST), and creatinine. The intraoperative variables included operative time, cardiopulmonary bypass (CPB) time, aortic crossclamp time, and selective cerebral perfusion time. The postoperative indicators included duration of mechanical ventilation, intensive care unit (ICU) length of stay, spinal cord injury, low cardiac output syndrome, stroke, pulmonary complications, acute renal failure, gastrointestinal complications, hospitalization costs, and death.

Patients in cluster 1 had significantly more severe clinical symptoms and complications than patients in cluster 2. Therefore, we defined cluster 1 and cluster 2 as the ultra-high-risk group and the high-risk group, respectively.

### CTA findings

#### Profiles of CTA factors

There was no significant difference in the longitude length of AD (52.96 ± 7.96 cm vs. 52.03 ± 7.09 cm, *P* = 0.100) and AD% (98.95% ± 4.65% vs. 99.01% ± 3.98%, *P* = 0.279) between the ultra-high-risk and high-risk groups. Ascending aortic FL% was significantly higher in the ultra-high-risk group than in the high-risk group (71.91% ± 12.16% vs. 68.56% ± 12.10%, *P* = 0.001) (Table 4).

**Table 4:** Profiles of CTA factors.

Variable	Ultra-high-risk group (*n* = 109)	High-risk group (*n* = 235)	*P*-value
Initial rupture location			
Ascending aorta	68 (62.4%)	128 (54.5%)	0.168
Aortic arch	24 (22.0%)	48 (20.4%)	0.735
Descending aorta	17 (15.6%)	59 (25.1%)	0.048
Ratio of intercostal arteries from false lumen	0.55 ± 0.28	0.51 ± 0.30	0.279
False lumen thrombosis	63 (57.8%)	13 (56.6%)	0.834
Length of aorta (cm)	53.52 ± 7.72	52.60 ± 7.12	0.234
Length of dissection (cm)	52.96 ± 7.96	52.03 ± 7.09	0.100
AD%	98.95% ± 4.65%	99.01% ± 3.98%	0.279
FL%			
Ascending aorta	71.91% ± 12.16%	68.56% ± 12.10%	0.001
Thoracic-descending aorta	69.05% ± 10.54%	68.06% ± 8.55%	0.242
Descending aorta	69.81% ± 9.82%	68.82% ± 8.25%	0.105
Abdominal aorta			
Celiac trunk	66.34% ± 11.60%	64.36% ± 8.67%	0.099
Right renal artery	63.24% ± 8.17%	62.99% ± 7.15%	0.267

#### Branch artery perfusion status

With reference to the branch perfusion classification proposed by Sukgu and the Penn classification, the branch artery perfusion status is presented in Table 5. The branch artery perfusion status was worse with right coronary artery (*P* = 0.022), innominate artery (*P* = 0.022), right renal artery (*P* = 0.011), and left renal artery (*P* = 0.027) in the ultra-high-risk group than in the high-risk group.

**Table 5:** Branch artery perfusion status.

Variable	Ultra-high-risk group (*n* = 109)	High-risk group (*n* = 235)	*P*-value
Left coronary artery			0.294
True lumen	102 (93.6%)	226 (96.2%)	0.288
True lumen and false lumen	7 (6.4%)	8 (3.4%)	0.321
False lumen	0	1 (0.4%)	0.683
Right coronary artery			0.022
True lumen	54 (49.5%)	147 (62.6%)	0.023
True lumen and false lumen	44 (40.4%)	72 (30.6%)	0.076
False lumen	11 (10.1%)	16 (6.8%)	0.292
Innominate artery			0.022
True lumen	42 (38.9%)	123 (52.3%)	0.021
True lumen and false lumen	63 (58.3%)	107 (45.5%)	0.034
False lumen	3 (2.8%)	5 (2.1%)	0.715
Left carotid artery			0.265
True lumen	67 (61.5%)	159 (67.7%)	0.260
True lumen and false lumen	41 (37.6%)	74 (31.5%)	0.263
False lumen	1 (0.9%)	2 (0.9%)	>0.999
Left subclavian artery			0.776
True lumen	68 (62.4%)	143 (60.9%)	0.786
True lumen and false lumen	39 (35.8%)	87 (37.0%)	0.824
False lumen	2 (1.8%)	5 (2.1%)	0.857
Celiac trunk			0.208
True lumen	60 (55.0%)	144 (61.5%)	0.254
True lumen and false lumen	34 (31.2%)	67 (28.6%)	0.471
False lumen	15 (13.8%)	23 (9.8%)	0.280
Superior mesenteric artery			0.279
True lumen	77 (70.6%)	177 (76.0%)	0.294
True lumen and false lumen	30 (27.5%)	54 (23.2%)	0.361
False lumen	2 (1.8%)	2 (0.9%)	0.808
Right renal artery			0.305
True lumen	73 (67.0%)	173 (74.2%)	0.163
True lumen and false lumen	24 (22.0%)	27 (11.6%)	0.011
False lumen	12 (11.0%)	33 (14.2%)	0.421
Left renal artery			0.637
True lumen	56 (51.4%)	134 (57.5%)	0.287
True lumen and false lumen	32 (29.4%)	44 (18.9%)	0.027
False lumen	21 (19.3%)	5 5 (23.6%)	0.368
Right common iliac artery			0.059
True lumen	58 (53.2%)	139 (59.9%)	0.243
True lumen and false lumen	48 (44.0%)	89 (38.4%)	0.277
False lumen	3 (2.8%)	4 (1.7%)	0.830
Left common iliac artery			0.226
True lumen	46 (42.2%)	121 (52.2%)	0.086
True lumen and false lumen	60 (55.0%)	110 (47.4%)	0.155
False lumen	3 (2.8%)	4 (1.7%)	0.830

#### Significant factors related to the severity of acute DeBakey type I aortic dissection

In multivariate binary logistic regression analysis, the risk factors of CTA for the severity of acute DeBakey type I aortic dissection in patients undergoing FET and total arch replacement (ultra-high-risk or high-risk) were ascending aortic FL% [odds ratio (OR), 11.929; 95% confidence interval (CI), 1.421–100.111; *P* = 0.022], location of initial tear (OR, 0.678; 95% CI, 0.468–0.984; *P* = 0.041), degree of left iliac artery involvement (OR, 1.948; 95% CI, 1.150–3.299; *P* = 0.013), and degree of right coronary artery involvement (OR, 1.461; 95% CI, 1.009–2.117; *P* = 0.045).

The AUROC value of this multivariate logistic regression analysis for the severity of acute DeBakey type I aortic dissection in patients undergoing FET and total arch replacement was 0.940 (95% CI: 0.914–0.967; *P* < 0.001). The AUROC value of the four risk factors were as follows: ascending aortic FL% with 0.841 (95% CI: 0.798–0.884; *P* < 0.001), location of initial tear with 0.649 (95% CI: 0.294–0.409; *P* < 0.001), degree of left iliac artery involvement with 0.602 (95% CI: 0.537–0.668; *P* = 0.002), and degree of right coronary artery involvement with 0.742 (95% CI: 0.683–0.800; *P* < 0.001).

## Discussion

In our study, we used the ratio of the arch length of the false lumen as a substitute for the ratio of diameter, area, or volume to evaluate the extent of the DeBakey type I aortic dissection. The main reasons are as follows. First, the shapes of the true and false lumens are irregular. Therefore, the diameters of the true and false lumens cannot represent their true size. Second, the intimal flap constantly oscillates during systole and diastole. Third, without the use of electrocardiogram (ECG) gating, it is difficult to measure the diameter, area, or volume because of the artifact caused by intimal flap oscillation. Although aortic CTA is performed with ECG gating, the whole aorta may not be in the same cardiac phase. Fourth, the two hinge points of the intimal tear are relatively fixed in all phases of the cardiac cycle. And the arch lengths of the false lumen and true lumen change simultaneously. The ratio of arch lengths is less affected by the cardiac cycle. Finally, the arch length ratio is easier to measure.

Acute DeBakey type I aortic dissection is associated with significant morbidity and mortality. With advances in surgical techniques and postoperative care, most patients with acute DeBakey type I aortic dissection have an excellent prognosis (14, 15). Therefore, early diagnosis and treatment of DeBakey type I aortic dissection is crucial (16). At our institution, patients with acute DeBakey type I aortic dissection are operated on at the earliest opportunity. The methods used include surgical and hybrid therapies (17, 18), including surgery of the aortic root, ascending aorta, and aortic arch. Patients with aortic dissection limited to the ascending aorta (without involvement of the aortic arch, DeBakey type II) and patients undergoing a hybrid procedure are in small proportions at our center. To reduce surgical procedure bias, patients undergoing combined FET and total arch replacement were included in this study.

The results showed that the location of the initial tear was one of the risk factors (OR, 0.678; *P* = 0.041). The location of initial tear factor was a ranking variable. The locations of the initial tear in the ascending aorta, aortic arch, and descending aorta were ranked 1, 2, and 3, respectively. Thus, patients with an initial tear in the ascending aorta were considered to be more severely affected than those with an initial tear in the aortic arch or descending aorta. In the reports by Yutaka Okita et al. (19), the early mortality rate of retrograde DeBakey type I aortic dissection was more favorable than that of antegrade DeBakey type I aortic dissection. The reason may be that it was easier to induce coronary malperfusion or aortic valve problems in patients with antegrade DeBakey type I aortic dissection than in patients with retrograde DeBakey type I aortic dissection.

According to the results of this study, the degree of right coronary artery involvement (OR, 1.461; *P* = 0.045) and the degree of left iliac artery involvement (OR, 1.948; *P* = 0.013) on preoperative aortic CTA were the risk factors considered for the severity of acute DeBakey type I aortic dissection undergoing FET and total arch replacement. The status of branch artery perfusion is an important indicator of DeBakey type I aortic dissection. Malperfusion is defined as a consequence of aortic dissection caused by aortic branch arteries with compromised blood flow resulting in ischemic dysfunction of one or more organs (20). The incidence of patients presenting with any type of organ malperfusion in our study was 15.4% (53/344). It was higher in the study of 2,137 consecutive patients with acute type A aortic dissection included in the German registry, and the incidence of malperfusion was 33.6% (717/2,137) (21). Malperfusion is associated with severe adverse outcomes in patients with acute DeBakey type I aortic dissection (22, 23). It is suggested that there should be an individualized therapeutic strategy to resolve malperfusion. Therapeutic strategies include optimal positioning for arterial cannulation, combined surgery, and postoperative care. Combined surgery with coronary artery bypass grafting or extra-anatomic bypass has been widely used to correct the phenomenon of organ malperfusion.

The values of the ratio of FL arch length to aortic circumference in the five aortic levels were measured on preoperative aortic CTA. Ascending aortic FL% was significantly higher in the ultra-high-risk group than in the high-risk group (71.91% ± 12.16% vs. 68.56% ± 12.10%, *P* = 0.001) and was the risk factor of preoperative aortic CTA considered for the severity of acute DeBakey type I aortic dissection (OR, 11.929; *P* = 0.022). The AUROC value of the ascending aortic FL% was 0.841 (95% CI: 0.798–0.884; *P* < 0.001) for the severity of acute DeBakey type I aortic dissection in patients undergoing FET and total arch replacement. The result showed that the factor of ascending aortic FL% is capable of predicting the severity of acute DeBakey type I aortic dissection patients in patients undergoing FET and total arch replacement. However, FL% of other aortic levels was not the risk factor for the severity of acute DeBakey type I aortic dissection.

## Limitations of the study

The present study had several limitations. First, the patients with severe DeBakey type I aortic dissection that died during transfer or while awaiting surgical repair in the emergency department were not included in this study. In addition, we tried to use cluster analysis to divide the patients into two severity groups. And the result of the two severity grade groups was used as the binary dependent variable in the logistic model. Finally, this study only represents our single-center experience with a single procedure. Larger multicenter studies are recommended to validate the risk factor of FL% of preoperative aortic CTA for the severity of acute DeBakey type I aortic dissection in the future.

## Conclusion

Ascending aortic FL% was validated as an essential radiologic index for assessing the severity of acute DeBakey type I aortic dissection in patients undergoing FET and total arch replacement. Higher values of ascending aortic FL% were more severe. In preoperative aortic CTA of patients with aortic dissection, the ratio of FL arch length to aortic circumference may be a reliable and useful parameter and deserves further investigation.

## Data Availability

The original contributions presented in the study are included in the article/Supplementary Material, further inquiries can be directed to the corresponding authors.
