# Supplementary material for: FL% is associated with the severity of acute DeBakey type I aortic dissection in patients undergoing frozen elephant trunk and total arch replacement
Source: Front Surg. 2024 Apr 9;11:1329771. doi: 10.3389/fsurg.2024.1329771 (PMC11035816; doi:10.3389/fsurg.2024.1329771)
Supplement: Supplementary file 1 [file Table1.docx]

**Supplementary TABLE 1. Risk factors of preoperative CTA for the severity of acute DeBakey type A acrtic Dissection**

| **Variable** | **OR** | **95% CI** | **P value** |
| --- | --- | --- | --- |
| Ascending aortic FL% | 11.929 | 1.421-100.111 | .022 |
| False lumen thrombosis | 1.526 | 0.845-2.754 | .161 |
| Initial rupture location | 0.678 | 0.468-0.984 | .041 |
| *Branch artery perfusion status* |  |  |  |
| Right coronary artery | 1.461 | 1.009-2.117 | .045 |
| Innominate artery | 1.575 | 0.941-2.635 | .084 |
| Left subclavian artery | 0.736 | 0.455-1.189 | .210 |
| Left renal artery | 0.792 | 0.564-1.112 | .179 |
| Left common iliac artery | 1.948 | 1.150-3.299 | .013 |

CI, confidence interval; OR, Odds ratio.
